# Reassessing the Host Defense Peptide Landscape

**DOI:** 10.3389/fchem.2019.00043

**Published:** 2019-02-04

**Authors:** Evan F. Haney, Suzana K. Straus, Robert E. W. Hancock

**Affiliations:** ^1^Centre for Microbial Diseases and Immunity Research, University of British Columbia, Vancouver, BC, Canada; ^2^Department of Chemistry, University of British Columbia, Vancouver, BC, Canada

**Keywords:** antimicrobial peptide, antibiofilm peptide, host defense peptide, chemical space, peptide therapeutics

## Abstract

Current research has demonstrated that small cationic amphipathic peptides have strong potential not only as antimicrobials, but also as antibiofilm agents, immune modulators, and anti-inflammatories. Although traditionally termed antimicrobial peptides (AMPs) these additional roles have prompted a shift in terminology to use the broader term host defense peptides (HDPs) to capture the multi-functional nature of these molecules. In this review, we critically examined the role of AMPs and HDPs in infectious diseases and inflammation. It is generally accepted that HDPs are multi-faceted mediators of a wide range of biological processes, with individual activities dependent on their polypeptide sequence. In this context, we explore the concept of chemical space as it applies to HDPs and hypothesize that the various functions and activities of this class of molecule exist on independent but overlapping activity landscapes. Finally, we outline several emerging functions and roles of HDPs and highlight how an improved understanding of these processes can potentially be leveraged to more fully realize the therapeutic promise of HDPs.

## Introduction

Driven by the emergence of antibiotic resistance throughout the world and a dearth of antimicrobials in the drug development pipeline, we are on the precipice of returning to a pre-antibiotic age (Martens and Demain, 2017). Since their discovery in the 1980s, antimicrobial peptides (AMPs), naturally occurring polypeptide sequences (~12–50 residues) comprised of cationic and hydrophobic amino acids with direct antibacterial activity (Hancock and Sahl, 2006; Nguyen et al., 2011; Fjell et al., 2012), have long been touted as one solution to this impending medical crisis. Statements such as “promising alternative to antibiotics,” “potential to address to growing problem of antibiotic resistance” and “hold promise to be developed as novel antibiotics” appear in almost every research article describing novel AMP sequences. In fact, the vast majority of studies related to AMPs have sought to identify and characterize peptides with potent and broad spectrum antimicrobial properties. Common strategies involve searching for novel peptides from natural sources either through the analysis of increasingly-exotic biological organisms and tissue extracts (Kim et al., 2018), identifying potential AMP sequences from genomic sequence information (Rodríguez-Decuadro et al., 2018; Yang et al., 2018), or excising predicted antimicrobial sequences from larger proteins (Pane et al., 2016; Abdillahi et al., 2018). Furthermore, a large portion of the relevant scientific literature is devoted to studies aimed at selectively enhancing the antibacterial potency of synthetic peptides either by systematically altering the amino acid composition of natural AMPs (Akbari et al., 2018; Chen et al., 2018) or designing novel sequences based on the structural and biophysical properties of known AMPs (Haney and Hancock, 2013; Kumar et al., 2018). Indeed, the prospect of finding a peptide with broad spectrum antimicrobial activity toward antibiotic resistant pathogens that plague human populations is a worthy endeavor that has captured the imagination and resources of many scientific research groups worldwide.

In spite of their tremendous promise, no peptide-based antibiotic has to-date realized regulatory approval (although several are in advanced clinical trials). There are many potential reasons for this apparent lack of success in developing this class of molecules as medicines, including low stability, toxicity, and high cost-of-goods (Haney and Hancock, 2013). However, it may be that we have already achieved the limits of antimicrobial potency for AMPs, through either natural selection by evolutionary processes or rational design, and that AMPs may never be able to achieve the same clinical outcomes as conventional antibiotics. Even more troubling is the possibility that the direct antibacterial effects of AMPs may not represent the primary biological functions of these molecules in nature and that researchers could be spending considerable effort searching for an elusive “optimal” AMP sequence that may not exist. For instance, it is well-established that the direct antibacterial activity of most AMPs is dramatically reduced under physiological conditions that would be encountered *in vivo* (Goldman et al., 1997; Bowdish et al., 2005; Starr et al., 2016). Consequently, it may be that the *in vitro* screening procedures employed to date do not effectively capture the true potential of this class of molecule since a growing amount of *in vivo* data has demonstrated the efficacy of AMPs in various animal models related to infection and inflammation, further underscoring their tremendous clinical potential. Indeed, over that past decade or so, we have begun to appreciate the other biological functions that can be inherent to amphipathic cationic peptides. These include such activities as immune modulation, including anti-infective (e.g., immune cell recruitment), anti-inflammatory, and wound healing properties, as well as antibiofilm activity. To emphasize the multifaceted nature of these cationic polypeptides, the term “Host Defense Peptide” (HDP) (Nijnik and Hancock, 2009; Takahashi et al., 2010) is now more commonly used to encompass the breadth of biological processes that are influenced by these versatile biomolecules, although the terms AMP and antibiofilm peptides are still accurate when considering only activities against planktonic and biofilm cells, respectively.

Our goal in this review is to question some of the most tightly held beliefs regarding the natural roles and functional potentials of AMPs and HDPs. We begin by critically examining the purported mechanism of action of AMPs as directly targeting the membrane of bacterial cells and highlight some of the advances that have helped many push beyond simplified models of antimicrobial activity. We then argue the need to shift the paradigm from appreciating these molecules as direct acting antibacterial compounds toward multi-faceted mediators of a wide range of biological processes. In particular, we explore the concept of chemical space (all possible polypeptide sequences of a given length) in the context of HDPs and postulate that the activity landscapes related to each biological function of HDPs are distinct, but overlapping. Finally, we outline several emerging roles of HDPs in relation to health and disease and highlight some of the new and exciting work being done to fully realize the therapeutic promise of HDPs.

## Mechanism of AMPs Activity—A Complex Question

For many years now, AMPs have largely been investigated in the context of their ability to kill bacteria by disrupting membranes ultimately leading to cell lysis and death (Hancock and Sahl, 2006; Zasloff, 2009; Kumar et al., 2018; Lázár et al., 2018). Experts in the field have often discussed at length the exact nature of the membrane perturbation, i.e., whether the peptides kill bacteria through transmembrane pore (Rapaport and Shai, 1991; Matsuzaki et al., 1998) or non-pore (Shai, 2002; Yeaman and Yount, 2003; Lee et al., 2016) mechanisms. Alternatively, the effect of bilayer integrity may be compromised upon reaching a certain threshold peptide concentration at the membrane surface (Andersson et al., 2016; Epand et al., 2016). The main models presented include the barrel-stave, carpet, detergent, toroidal pore, aggregate (Hale and Hancock, 2007), and electroporation (Lee et al., 2016) models, or combinations thereof (Kumar et al., 2018), and these have been extensively discussed in other reviews. Many detailed studies have relied on the use of a handful of biophysical methods to determine how these peptides perturb membranes (Okada and Natori, 1983; Zasloff, 1987; Lehrer et al., 1989; Arias et al., 2018; Marquette and Bechinger, 2018). Aspects considered in these studies include the structure of the peptide in the membrane, the insertion and interaction of the peptide into model lipid bilayers, lipid selectivity and/or ability to cause leakage. While all of these observations are valid within the context of the experimental setup, in the following section, we will examine how these findings may not be relevant to how HDPs actually kill bacteria outside of a culture tube. This is particularly relevant for AMPs that lack membranolytic activity. Specifically, we critically examine several mechanistic principles that are often generalized for AMPs and discuss how researchers have begun to unravel varied and complex mechanisms of action for this class of peptides.

### Does the Electrostatic Attraction Between AMPs and Membranes Dictate Activity?

Most antimicrobial peptides are cationic and amphipathic with a net charge ranging from +2 to +9, due to an abundance of Arg and Lys residues within their sequence (Haney and Hancock, 2013). Given that the bacterial cytoplasmic membrane contains a high proportion of phospholipids with negatively charged headgroups [e.g., phosphatidylglycerol (PG) and cardiolipin (CL)], the initial interaction between a peptide and a bacterial cell is generally considered to be electrostatic in nature followed by an association of the hydrophobic domains of AMPs with the hydrophobic core of membranes. In reality, the path between a cationic peptide and the anionic cytoplasmic membrane is fraught with potential peptide binding targets and littered with bacterial cell surface components that necessitate this process should be more than a simple electrostatic interaction.

For instance, Gram-negative bacteria possess an outer membrane which acts as a selective barrier and protects the cell from the action of various antibiotics. Furthermore, lipopolysaccharides (LPS) are present in high abundance on the surface of the outer membrane of Gram-negative bacteria. LPS molecules bear multiple negative charges that are typically neutralized by the presence of divalent cations (e.g., Mg^2+^ and Ca^2+^) which in turn stabilize the outer membrane (Vaara, 1992). LPS offers an electrostatic and hydrophobic binding partner for approaching cationic amphipathic peptides (which have a higher affinity for LPS than the native divalent cations) and upon membrane binding, the peptides competitively displace the divalent cations which subsequently interferes with lipid packing and leads to increased permeability of the outer membrane thereby mediating their so-called self-promoted uptake (Hancock, 2001). Beyond this, it is unclear what the driving force is that allows peptides to transition from the Gram-negative outer membrane to the surface of the cytoplasmic membrane and beyond. Conceivably, this could involve some combination of peptide concentration gradients, Donnan potentials (created by the presence of polyanionic membrane-derived oligosaccharides in the bacterial periplasm) or the electrical potential gradient across the cytoplasmic membrane (Nikaido, 2003).

In Gram-positive bacteria, a thick layer of peptidoglycan surrounds the bacterial cell and provides structural support. An AMP should transit quite freely through the netlike structure of a Gram-positive cell wall to interact with the cytoplasmic membrane (Vollmer and Bertsche, 2008). However, while peptidoglycan itself lacks an explicit negative charge, teichoic acid, and lipoteichoic acids can be found interspersed throughout the peptidoglycan structure and confer a surface negative charge. These anionic molecules also provide binding sites for HDPs (Scott et al., 1999a), and these would presumably need to be circumvented to reach the cytoplasmic membrane.

Since the electrostatic attraction between a cationic AMP and the anionic bacterial cell surface is considered essential to the overall mechanism of action, several studies have examined how charge relates to activity (Dathe et al., 2001; Mihajlovic and Lazaridis, 2012) and whether this property could be manipulated to improve antibacterial potency and selectivity for bacterial cells. Results from these studies suggest that there is an optimal charge/hydrophobicity balance needed to ensure equilibrium between activity and toxicity. For example, increasing the charge of magainin 2 from +3 to +5 improved the antibacterial activity against both Gram-positive and Gram-negative bacteria, but an increase to +6 or +7 led to increased hemolytic activity and loss of antimicrobial potency (Dathe et al., 2001). It has been suggested that the loss of activity in highly cationic peptides may be due to the fact that this would result in an extremely strong interaction between the peptide and the anionic phospholipid headgroups that would prevent translocation of the peptide into the inner leaflet of the membrane (Yeaman and Yount, 2003). Alternatively, the increased charge may perturb the kinetic network, i.e., the careful balance between peptide-bacteria interactions vs. peptide-host cell interactions (Starr et al., 2016).

In addition to overall charge on a peptide surface, the specific location of the charged residues (and by extension the hydrophobic residues) along the length of a peptide has a significant influence on antibacterial potency and toxicity (Hilpert et al., 2006; Leptihn et al., 2010; Archer et al., 2011; Yin et al., 2012; Hollmann et al., 2016). The fact that even a single amino acid change within an AMP sequence can dramatically alter the antibacterial and/or toxicity profile of a peptide would suggest that the influence of biophysical parameters such as charge and hydrophobicity are important within the context of the AMP sequence in question. Therefore, sequence manipulations aimed at improving potency may be difficult to apply broadly to all AMPs in general.

Finally, another indication that positive charge may not play the defining role in determining AMP potency is the fact that several anionic antimicrobial peptides have also been reported in the literature (Harris et al., 2009) and many of these adopt amphipathic structures and can interact with membranes, akin to the interactions that have been observed for cationic AMPs.

### Do All Antimicrobial Peptides Act to Destroy Bacterial Membranes?

Since AMPs are known to interact with phospholipid bilayers, it was originally claimed that all AMPs act as membrane disrupters in keeping with early studies that demonstrated that AMPs permeabilized membranes, e.g., of vesicles. However, most of those studies relied on data obtained at concentrations far above the minimal inhibitory concentration (MIC), or under artificial assay conditions using model membranes and very high peptide:lipid ratios relative to the conditions that would occur during killing of bacterial cells (Wu et al., 1999; Wimley, 2010).

While it is true that AMPs must interact with bacterial membranes as part of their overall mechanism of action, this dogma of membrane targeting leading to lysis or cytoplasmic leakage has now been effectively refuted as summarized previously (Hancock and Sahl, 2006; Fjell et al., 2012). Indeed it is now well-understood that in addition to membrane interactions, many AMPs act on membrane-associated targets (e.g., cell wall biosynthesis, cell division, etc.) or on cytoplasmic targets (e.g., macromolecular synthesis in cells, heat shock proteins, etc.; Otvos, 2005; Hale and Hancock, 2007; Fjell et al., 2012). One of the earliest examples of this phenomenon was buforin II, a histone derived AMP from Asian toads (Park et al., 1998). Interestingly, *E.coli* cells treated with buforin II were not lysed, even at 5X the MIC, and this peptide did not perturb model membranes, clearly demonstrating that membrane perturbation does not contribute to the bactericidal effect. Instead, it was demonstrated that this peptide translocated into the bacterial cytoplasm where it readily bound DNA and RNA, implicating this interaction in the mechanism of action (Park et al., 1998). Subsequently, Wu et al. described a broad range of peptides that did not completely depolarize bacterial cells at their MIC (Wu et al., 1999). Alternatively, human α-defensin 5 has been shown to translocate into the cytoplasm of *E. coli* where it accumulates at the cell division plate and at opposite poles of the cell, suggesting that part of the antibacterial mechanism of this AMP is due to interference with cellular division processes (Chileveru et al., 2015). Cell division targeting through QueE was also recently shown for peptide C18G, a synthetic AMP derived from platelet factor IV (Yadavalli et al., 2016). Other peptides interfere with membrane associated processes, such as binding to lipid II which is involved in cell wall and teichoic acid biosynthesis, thereby inhibiting cell wall biogenesis (Wiedemann et al., 2001; Sass et al., 2010; Schneider et al., 2010). Several more examples of AMPs that target intracellular bacterial components exist, including: PR-39 (Boman et al., 1993), indolicidin (Subbalakshmi and Sitaram, 1998), pyrrhocoricin (Kragol et al., 2001; Taniguchi et al., 2016), human β-defensin 4 (Sharma and Nagaraj, 2015), proline-rich AMPs (Scocchi et al., 2011; Li et al., 2014; Florin et al., 2017), and many others (Hale and Hancock, 2007; Shah et al., 2016).

Indeed it has been proposed that AMPs are likely to have multiple modes of action, a feature that has earned them the moniker of “dirty drugs” (Friedrich et al., 2001; Hancock and Sahl, 2006). Adding to this complexity, it has been proposed that individual AMPs elicit a unique bacterial response which was demonstrated by challenging *E. coli* with four physico-chemically related peptides: magainin 2, pleurocidin, buforin II, and a synthetic D-amino acid peptide D-LAK120-AP13 (Kozlowska et al., 2014). In this case, treatment of *E. coli* with sub-lethal concentrations of each peptide caused both metabolic and gene expression changes that were unique to each peptide, suggesting that every AMP employs a unique mechanism of action to exert their antibacterial effects. Several other studies have demonstrated that AMP treatment causes substantial changes to overall gene expression profiles (Bader et al., 2003; Tomasinsig et al., 2004; Overhage et al., 2008; Majchrzykiewicz et al., 2010; Le et al., 2016; Nagarajan et al., 2018), further demonstrating the complexity of the bacterial response to natural and synthetic AMPs.

As stated above, interactions between AMPs and biological membranes occur and these interactions play a key role in the overall mechanism of action for this class of molecules. It seems possible that some AMPs, like magainin (Matsuzaki, 1998) and/or melittin (van den Bogaart et al., 2008), exert their primary antibacterial (and/or cytotoxic) effects through a lytic mechanism of action. However, applying the concept of membrane disruption to all AMPs is likely an oversimplification of a complex and dynamic process. As work continues in the field of AMP research it will be necessary to press beyond these simplified models that are often invoked to explain the mechanistic details underpinning the biological functions of AMPs.

### Do AMPs Adopt a Specific “Active” Conformation?

Another frequently-characterized feature of AMPs is their ability to fold into a variety of secondary structures including α-helices, β-structures, turns, extended structures and other permutations (Nguyen et al., 2011). It is generally accepted that most linear AMPs are unstructured in aqueous solutions and undergo a conformational change to a folded state as they bind and insert into biological membranes. Since this membrane interaction is required for the antibacterial effects of AMPs (even if the target is intracellular, the peptide must cross bacterial membranes), in the past it was considered that this membrane bound structure represented the “active” conformation. Much has been written about peptide interactions with membranes, with some authors suggesting formal channels (barrel-stave, toroidal pore models) while others have suggested more casual interactions (carpet, detergent, and aggregate model; Lee et al., 2016). However, considering the above-described diversity of mechanisms, ultimately the most satisfying models would describe how some peptides are able to translocate across membranes without lethally permeabilizing them. This is a known feature of immunomodulatory HDPs that must translocate into cells to mediate their activities (Lau et al., 2005; Mookherjee et al., 2009) and such peptides fall into the general class of cell penetrating peptides (Sandgren et al., 2004; Zorko and Langel, 2005; Guidotti et al., 2017). That AMPs do the same is suggested by demonstrations that some peptides can accumulate in the cytoplasm of bacteria (Park et al., 1998; Powers et al., 2006) or are readily taken up by eukaryotic cells (Tomasinsig et al., 2006).

Moreover, several studies have examined how AMP sequences correlate to peptide structure and how this may be related to antibacterial potency. For instance, aurein 2.2 and 2.3 are natural cationic AMPs from the frog *Litoria aurea* (Rozek et al., 2000). They are both 16 amino acid residues in length, have a net +2 charge, and an amidated C-terminus. Circular dichroism and NMR studies have shown that both peptides adopt a continuous α-helical structure in a membrane or membrane-mimetic environment (Pan et al., 2007; Cheng et al., 2009, 2010, 2011). This structure is only present when the peptides interact with the membrane, hence it could be assumed to be important for function. However, an analog of aurein 2.3 with a carboxylated C-terminus also adopts the same structure as the natural form, but does not have any antimicrobial activity (Pan et al., 2007).

The example cited above suggests that AMPs do not necessarily adopt a specific “active” conformation, i.e., there is no direct correlation between the amount or type of secondary structure and any quantifiable biological activity such as MIC. Indeed, short polypeptides are notorious for their conformational flexibility and several examples of natural and synthetic AMPs with a high degree of structural plasticity have been reported in the literature including: 1018 (Wieczorek et al., 2010), indolicidin (Nagpal et al., 2009), HHC-36 (Nichols et al., 2013), Gad-2 (McDonald et al., 2015) etc. It is this structural plasticity that makes peptides natural biological messengers (Henninot et al., 2018) and this flexibility in structure likely ensures that interactions between AMPs and their targets are malleable, enabling them to interact with a variety of binding targets including not only membranes, but also DNA, RNA, and certain proteins. These interactions, in turn, ensure that AMPs and HDPs are active against a broad range of microorganisms (including their biofilm growth states) while also being capable of causing pleiotropic effects in the host, all of which are essential to innate host defense processes (Hancock and Sahl, 2006, 2013).

### Are Bacteria Able to Develop Resistance to AMPs?

As natural molecules involved in host defense, HDPs have co-evolved for millions of years alongside bacteria and it has been frequently argued that bacteria are virtually incapable of developing resistance to AMPs. This is often touted as one of the attractive features of developing synthetic AMPs as alternatives to antibiotics. Unfortunately, bacteria are quite resourceful and indeed several resistance mechanisms to AMPs have been reported (Nizet, 2006; Bechinger and Gorr, 2017). Examples include remodeling of the membrane to reduce the overall negative charge, blocking the anionic groups in LPS by attaching an aminoarabinose group or decorating TA polymers with D-alanine moieties to counteract the negative charge arising from the phosphate groups in the TA monomers. Furthermore, AMPs may be degraded by the action of bacterial proteases (Sieprawska-Lupa et al., 2004; Lai et al., 2007) or they may simply be expelled from the cell following upregulation of bacterial efflux systems (Joo et al., 2016). Alternatively, the ability of peptides to induce resistance regulons in *Pseudomonas* to some extent dictated their activity against this bacterium (McPhee et al., 2003; Fernández et al., 2012). Regardless of which resistance mechanism is invoked by a particular bacterial species, it is important to consider these resistance mechanisms as we continue the search for novel AMP sequences with ever increasing antibacterial potency. At best, the various resistance mechanisms described for AMPs indicate that any new peptide-based antibiotic, once introduced in the clinic, would be prone to similar patterns of resistance as those observed for conventional antibiotics (Blair et al., 2015). In this scenario, AMPs could be viewed as merely stemming the rising tide of antibiotic resistance rather than acting as a miracle drug that will solve all our problems.

### Are Antibiofilm Peptides Distinct From AMPs?

The development of antibiofilm peptides and their potential to address the issues of biofilm-associated infections has been reviewed elsewhere (Batoni et al., 2016; de la Fuente-Núñez et al., 2016; Pletzer and Hancock, 2016) and hence, we will only briefly discuss some of their properties here. This class of peptide acts against biofilms formed by multiple species of bacteria, including the most resistant organisms in our society termed the ESKAPE pathogens (*Enterococcus faecium, S. aureus, Klebsiella pneumoniae, Acinetobacter baumannii, Pseudomonas aeruginosa, Enterobacter cloacae*) and other clinically relevant bacteria (de la Fuente-Núñez et al., 2014b). As mentioned above, structure activity relationship studies showed no direct overlap between antibiofilm and antimicrobial (vs. planktonic bacteria) activities. Thus peptides are able to inhibit biofilms formed by *Burkholderia cenocepacia* which is normally resistant to the effects of AMPs (Loutet and Valvano, 2011) and it is also possible to isolate peptides with excellent antibiofilm activity but poor activity against planktonic bacteria and vice versa (de la Fuente-Núñez et al., 2012). This suggests that the mechanism of action employed by antibiofilm peptides must be distinct from those employed by AMPs.

Recent work by our group has implicated the widespread bacterial stringent response as a common target for the antibiofilm activity of HDPs. When bacteria are subject to amino-acid starvation, fatty acid limitation, iron limitation, heat shock and other stressors (Crosse et al., 2000; Potrykus and Cashel, 2008), a stringent response is triggered through up-regulation of the two signaling nucleotides: guanosine tetraphosphate (ppGpp) and pentaphosphate (pppGpp) [collectively known as (p)ppGpp]. These signals cause the bacteria to divert nutrients from growth and division processes in order to promote survival, ultimately resulting in biofilm formation (Potrykus and Cashel, 2008; Wolz et al., 2010). In many bacterial species, ppGpp is required for biofilm growth and mutants lacking the enzymes responsible for generating (p)ppGpp are unable to elicit a stringent response and therefore do not form biofilms (Åberg et al., 2006; He et al., 2012; de la Fuente-Núñez et al., 2014b).

In this regard, the synthetic HDP (also termed Innate Defense Regulator or IDR) peptide IDR-1018 (de la Fuente-Núñez et al., 2014b), and several D-enantiomeric peptides, including DJK-5 (de la Fuente-Núñez et al., 2015), have been shown deplete (p)ppGpp from cells *in vivo*, as well as to directly interact with (p)ppGpp *in vitro*, by preferentially binding to it as compared to other nucleotides (e.g., GTP). In an *in vivo* mouse abscess model for which pathology (cutaneous lesion formation) is dependent on the stringent response, both peptides suppressed lesion formation by either *Staphylococcus aureus* or *Pseudomonas aeruginosa* (Mansour et al., 2016), and for the latter a specific role of the stringent response and suppression of the expression of the bifunctional (p)ppGpp metabolizing enzyme, SpoT, was indicated (Pletzer et al., 2017). A study showed that an analog of 1018, with its amino acid sequence reversed (Andresen et al., 2016), was equally able to co-precipitate ppGpp in a test tube and still exhibited inhibitory effects on *P. aeruginosa* biofilms. Similarly, the D-analog of this reversed sequence also depleted (p)ppGpp from cells (de la Fuente-Núñez et al., 2015). It is worth mentioning that Andresen et al. argued that since the reversed 1018 peptide sequence exhibited similar ppGpp and antibiofilm activities compared to the native 1018 peptide, that this could not explain the specificity of the mechanism of action or the involvement of the stringent response. We have recently addressed this critique in some detail (Pletzer et al., 2017) and we contend that binding of phosphorylated nucleotides may be a common feature of many cationic HDPs and represents a molecular interaction that could be exploited if we could better understand the specific peptide requirements for (p)ppGpp binding. Nevertheless, the implication of this molecular mechanism for antibiofilm peptides is that they must be able to translocate across the membrane into bacteria in order to act on this intracellular nucleotide.

Since the effect of antibiofilm peptide activity in mice is to inhibit the formation of necrotic lesions, it is worth mentioning that DJK-5 strongly suppressed the production of alpha-type phenol soluble modulins (Mansour et al., 2016), which are stringently regulated cytotoxins that are also involved in biofilm structuring (Periasamy et al., 2012). Evidently, HDPs that exhibit antibiofilm activities can mediate a range of biological functions and exert their activities through a variety of mechanisms; indeed peptide 1018 also possesses potent immunomodulatory functions and works in a wide range of *in vivo* animal models of infection and inflammation (Mansour et al., 2015).

To this point, we have examined several of the commonly held beliefs regarding the antibacterial functions of AMPs and have discussed how the prevailing view of these molecules has shifted from being simple membrane destroyers to biomolecules that exert their antimicrobial effects by targeting a plethora of bacterial components using a variety of mechanisms. Indeed, several features of AMPs have emerged over the years as contributing factors to the observed antibacterial potency including charge, hydrophobicity, and structure. However, we should be cautious about extrapolating the observed effects of a single peptide to all AMPs in general as this oversimplifies many of these processes and fails to appreciate that each individual peptide sequence mediates a variety of functions independently. This multifaceted nature of AMPs and HDPs is reflected in the fact that these molecules have biological functions that extend beyond bacterial cells. In the following sections, we will examine some of the other activities that have been observed for HDPs *in vitro* and *in vivo* and we will further examine how an appreciation of these additional functions is shaping the future clinical and therapeutic applications of these biomolecules.

## Activity Landscapes of Host Defense Peptides

Beyond questioning tightly-held beliefs about the bacteriostatic and bactericidal nature of AMPs, one has to consider that many HDPs influence a wide range of biological functions *in vivo*. Other types of activities, in addition to various forms of immune modulation (Hancock et al., 2016) and antibiofilm activity (Pletzer and Hancock, 2016) are increasingly being appreciated for HDPs and include (but are not limited to): antiviral (Gwyer Findlay et al., 2013), antifungal (Weerden et al., 2013) antiparasitic (Mor, 2009), anticancer (Gaspar et al., 2013), wound healing (Mangoni et al., 2016), adjuvanticity for vaccines (Nicholls et al., 2010), and more recently they have been proposed as biomarkers for certain diseases (Silva et al., 2018). Each of these have been extensively reviewed elsewhere (see review articles referenced above) and these “alternative” activities of HDPs are rapidly gaining prominence as more investigators examine these diverse biological effects. These broad activity classes also present tremendous opportunities for researchers to identify and optimize natural and synthetic peptide sequences that are tailored for a specific biological function. However, the question remains, what represents an “optimal” HDP sequence?

Since their discovery in insects (Steiner et al., 1981), mammals (Ganz et al., 1985; Selsted et al., 1985a,b) and frogs (Zasloff, 1987), the majority of research endeavors in the HDP field have been focused on identifying, characterizing and optimizing peptide sequences for their direct antibacterial activity while limiting toxicity toward eukaryotic cells (often assessed as hemolysis of red blood cells). Indeed, up until the last decade or so, the holy grail of AMP research was a peptide with potent activity against a wide assortment of bacterial pathogens *in vitro*, while exhibiting no toxicity toward the cells of the host. In general, the scientific community has had remarkable success in searching for antimicrobial HDP sequences from natural sources, as evidenced by the current tally of nearly 3,000 sequences deposited in the Antimicrobial Peptide Database (APD, http://aps.unmc.edu/AP/main.php) (Wang et al., 2015a). Many more studies have sought to manipulate the biophysical characteristics of these natural HDP sequences to optimize synthetic peptides for their antibacterial effects (reviewed in Fjell et al., 2012). Optimization strategies such as these typically manipulate a few biophysical traits of a given peptide and evaluate the effects of substituting specific amino acids at various points within the parent sequence. Normally, these parameters involve some combination of cationic charge and hydrophobicity and a small library of ~5–10 peptides is generated based on a starting peptide scaffold. In most published examples, some derivatives exhibit moderately enhanced antimicrobial potency relative to the parent sequence, or perhaps reduced toxicity, and this is then used to justify the design approach. It is difficult to estimate how many synthetic peptides have been evaluated in studies such as these, although manually-curated databases of published HDPs count between 11,000 and 17,000 entries (Fan et al., 2016; Pirtskhalava et al., 2016). Based on our own experience (and the number of synthetic peptides in our laboratory freezers), as well as the fact that the search term “Antimicrobial Peptide” yields more than 300,000 hits in PubMed, we would venture to guess that the actual number of peptides that have been created and tested in labs is substantially higher.

In principle, the possible chemical space of HDPs can be represented mathematically by the equation 20^*n*^, which encompasses all possible permutations and combinations of the 20 naturally occurring amino acids for a peptide of length *n* (the problem becomes exceedingly complex if we start to consider the 700 or so non-natural amino acids, enantiomers, and peptidomimetic backbones). Since the primary structure of a peptide and how these fold in three-dimensions dictates the biological activity of any given peptide (Fjell et al., 2012), if the activities of all the peptides within this chemical space could be evaluated, it would be possible to unequivocally identify the best HDP for any given type of activity. Unfortunately, this scenario is virtually impossible as this chemical space becomes overwhelmingly large rather quickly as even a chemical space limited to peptides of 10 residues in length would include over 10 trillion sequences (Table 1). It may be possible to limit the chemical space of HDPs by focusing on specific amino acids generally considered important for HDP function such as cationic (Arg and Lys) and hydrophobic (Gly, Ala, Val, Ile, Leu, Phe, Tyr, and Trp) residues (Table 1). However, it should be noted that all 20 amino acids are represented within HDP sequences deposited in the APD (Wang et al., 2015a) and such a strategy might remove potentially active sequences from the overall chemical space.

**Table 1:** Number of possible peptide sequences encompassing the chemical space of peptides of a given length (*n*).

**Peptide length (*n*)**	**Number of peptides in the chemical space (20*^***n***^*)**	**Chemical space limited to cationic (2) and hydrophobic (8) residues ((2+8)*^***n***^*)**
2	400	100
3	8,000	1,000
4	160,000	10,000
5	3,200,000	100,000
6	64,000,000	1,000,000
7	1,280,000,000	10,000,000
8	25,600,000,000	100,000,000
9	512,000,000,000	1,000,000,000
10	10,240,000,000,000	10,000,000,000
20	1.05 × 10^26^	1 × 10^20^
30	1.07 × 10^39^	1 × 10^30^

**Figure 1:**
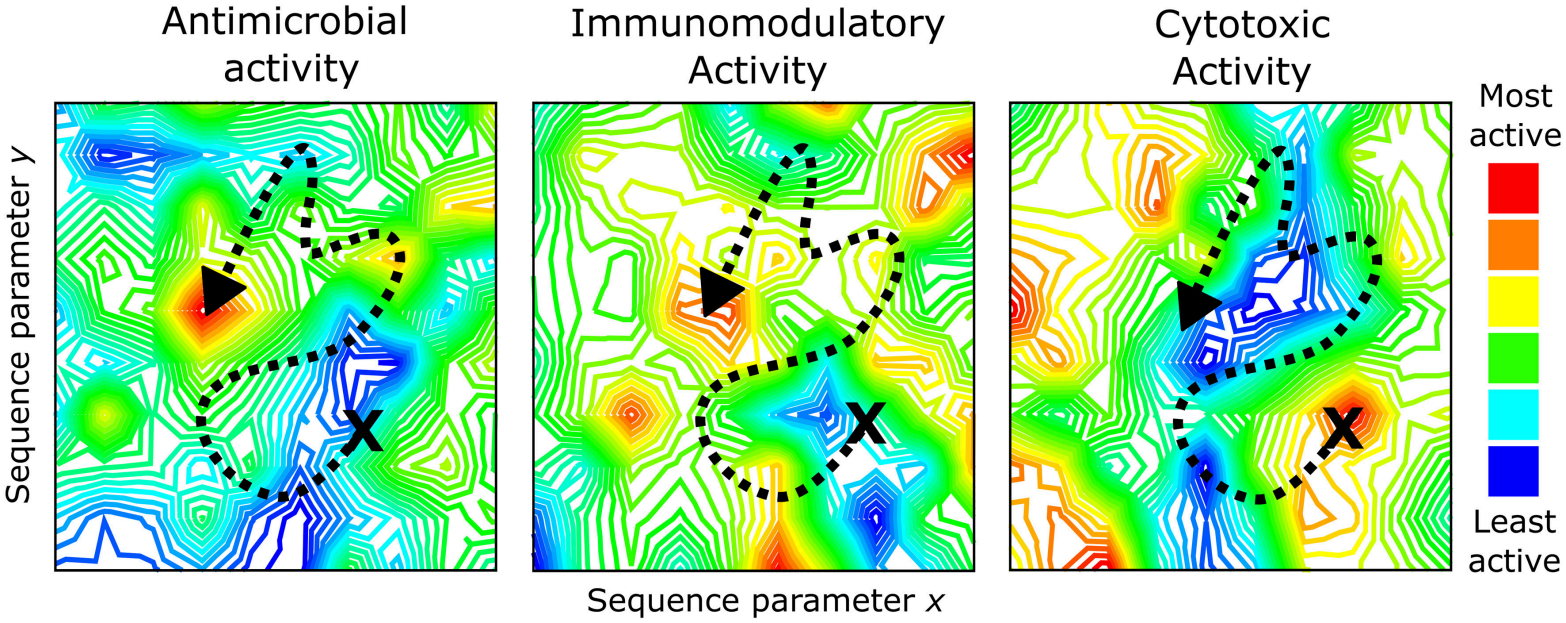
The activity landscapes for HDPs are complex (represented as topographical maps) and encompass a variety of biophysical characteristics such as charge, hydrophobicity, amphipathicity, folding propensity, etc. When optimizing synthetic peptides by moving around the chemical space of an activity of interest (represented by the dashed line), it is necessary to consider how this sequence alteration may impact other peptide properties and/or activities. This could result in a convergence of activities within an HDP sequence (e.g., antimicrobial and immunomodulatory activities above) or a reduction in one activity type (e.g., cytotoxicity landscape above). Topographical maps were generated by Contour Map Creator (http://contourmapcreator.urgr8.ch/), and the maps shown are only illustrative and actually correspond to various locations near Vancouver, Canada.

With such large numbers encompassing the possible chemical space of HDPs, it seems likely that the activities of individual peptides from within this chemical space would also be quite varied. An analogy that could be used to describe the activity landscape of this chemical space would be to envision this as a mountain range filled with peaks and valleys (Figure 1). Some peptides within the chemical space will have high activity (the peaks) while others will have low activity (the valleys). As we move around this chemical space by manipulating the primary amino acid sequence of a peptide and record the biological activities, this vast chemical space can be mapped with the ultimate goal of identifying the highest peak that represents a truly “optimal” sequence (e.g., left panel, Figure 1).

Such an approach might be more feasible if the activity landscape for each individual type of HDP activity were identical. However, there is ample evidence that these activity landscapes are independently defined for each biological function of HDPs. For instance, LL-37 possesses relatively weak direct antibacterial activity but inhibits *P. aeruginosa* biofilms at sub-inhibitory concentrations (Overhage et al., 2008). Selective antibiofilm activity by LL-37 has also been observed against *Aggregatibacter actinomycetemcomitans* which causes oral biofilms and can contribute to periodontal disease (Sol et al., 2013). In addition, several synthetic peptides have been identified with sub-inhibitory effects on bacterial biofilms including truncated variants of LL-37 (Luo et al., 2017) or the synthetic peptide WLBU2 (Lin et al., 2018). With respect to immunomodulatory activity, a synthetic HDP, IDR-1, offered protection in murine infection models against both Gram-positive or Gram-negative pathogens, despite the fact that IDR-1 exhibited no direct antibacterial effects *in vitro* (Scott et al., 2007). The selective modulation of the immune response by IDR-1 was found to be responsible for the protective effects, indicating that the antibacterial and immunomodulatory properties of HDPs were independently defined.

More recently, we sought to define the sequence requirements of two synthetic HDPs, IDR-1002 and IDR-HH2, to understand which residues contributed to the antibiofilm and immunomodulatory properties of these peptides (Haney et al., 2015). Using SPOT-synthesized peptide arrays, peptide libraries consisting of single amino acid substitution variants of the parent sequence were generated by replacing each residue with one of nine amino acids (R, K, D, G, A, I, L, V, or W). The antibiofilm and immunomodulatory (chemokine induction and anti-inflammatory) properties of each peptide variant were measured *in vitro* and plotted as substitution matrices to identify which residues contributed to each individual activity type. Interestingly, substantial overlap was observed between the activity profiles of the two peptides but there were also distinct residues that, when positionally substituted, appeared to preferentially improve one activity over another. These results imply that the activity landscapes for each biological function of HDPs within the chemical space are overlapping but distinct from each other (Figure 1). If these activity landscapes could be adequately defined for each activity type, it would, in principle, be possible to simultaneously optimize synthetic peptides for multiple activities, while avoiding potentially harmful sequences that are toxic or exert undesired effects. Therein lies the challenge for researchers working in the HDP field: how can we adequately define the activity landscape of the HDP chemical space?

Several approaches have been used to try and sample the chemical space of HDPs and get a glimpse into these activity landscapes, particularly for the antibacterial properties of HDPs. Early approaches involved screening of combinatorial peptide libraries to identify novel sequences with antibacterial activity (Blondelle and Houghten, 1996; Blondelle and Lohner, 2000) but these were effectively limited by the cost of such methods and the extreme numbers of variants such that only 6-mer sequences were considered.

Computational approaches have also been employed to design and optimize novel peptide sequences with enhanced antibacterial potency. Early attempts involved modeling AMPs as a language and using natural AMPs sequences to define a set of “grammars” that described the language (Loose et al., 2006) and the implementation of similar grammatical approaches continues to identify novel AMP sequences (Nagarajan et al., 2018; Porto et al., 2018). Other computational approaches have sought to establish quantitative structure activity relationships (QSAR) that model the activity of test peptides based on the chemical properties of AMPs using hundreds or thousands of so-called “descriptors” based on the primary structure and the physicochemical interrelationships of individual amino acids along the peptide chain. Artificial intelligence strategies (neural networks) were used to create models that quite accurately predicted the antibacterial activity of virtual peptides and ultimately identified novel 9-mer AMPs with enhanced antibacterial potency *in vitro* that were also effective in an *in vivo* model (Cherkasov et al., 2009). The emergence of machine learning methods to predict novel AMP sequences has proven quite popular and has made great strides in identifying many unexplored AMP sequences (Torrent et al., 2011; Maccari et al., 2013; Lee et al., 2017; Yoshida et al., 2018). Moving forward, an improved understanding of the mechanistic details of AMP activity coupled with the application of increasingly sophisticated computational algorithms will surely lead to more exciting outcomes from this line of inquiry.

Directed evolution methods have also been adapted to allow a specific biological interaction or biological activity to guide the discovery of novel peptide sequences. For instance, a phage display approach was used to identify peptides capable of binding to the cell surface of *E. coli* resulting in a novel antibacterial peptide sequence with activity against both *E. coli* and *P. aeruginosa*, although the resultant peptide was unfortunately only moderately active and did not inhibit other tested bacteria (Rao et al., 2013). A similar strategy was employed to identify AMPs with selective activity against *Listeria monocytogenes* (Flachbartova et al., 2016). Furthermore, phage display has successfully identified peptides with antiviral (Ojeda et al., 2016) and antifungal activity (de Oliveira et al., 2016) demonstrating the utility of such a technique to explore other activity landscapes within the chemical space of HDPs.

Recently, an elegant high-throughput synthetic biology approach was described wherein ~800,000 random 20mer peptide sequences were displayed on the surface of a bacterial cell as part of a fusion protein coupled to the outer membrane protein OmpA (Tucker et al., 2018). Pools of transformed bacterial cells before and after induction of the OmpA-peptide fusion constructs were sequenced and nearly 8,000 peptide sequences were identified as “hits” with potential antimicrobial activity. Of the 22 peptide sequences that were synthesized to validate the methodology, only two exhibited antibacterial activity when evaluated in the standard MIC assay using Mueller-Hinton broth but 18 (~80%) exhibited bactericidal activity when cells were treated in a simple tris-based buffer (10 mM Tris, 25 mM NaCl). It should be mentioned that the majority of these validation sequences were chosen based on opposite physico-chemical characteristics compared to classical AMPs (i.e., low hydrophobicity and neutral to negative charge) in an effort to sample unexplored regions of the peptide chemical space, while the two that exhibited the most potent activity conformed to properties of known AMPs. In fact, no particular bias toward hydrophobicity, charge, or enrichment of specific amino acids was observed for the ~8,000 “hit” sequences (Tucker et al., 2018), suggesting that the chemical composition of active antibacterial HDPs is likely more diverse than originally thought.

The examples described above primarily focused on sampling the activity landscape that defines the antibacterial properties of HDPs. However, the multifaceted nature of HDPs and their wide range of biological activities suggest that independent activity landscapes could be defined for every biological function of HDPs. In the following section, we examine some of these emerging roles of HDPs, beyond their direct antibacterial effects, that represent the next wave of research that could propel these molecules to clinical significance.

## HDPs in Health and Disease

### HDPs as Modulators of Microbial Communities

The drive to target pathogenic bacteria in the face of rising antibiotic resistance has spurred the lion's share of research into natural and synthetic HDPs. However, the interactions between bacterial cells and HDPs, involving the polycationic amphipathic peptides associating with polyanionic and hydrophobic surfaces, would not be limited to pathogenic bacteria, so it seems likely that an AMP would have similar antibacterial effects on commensal bacteria when present at sufficiently high concentrations. Inevitably, the disruption of the natural microbiota could lead to the expansion of opportunistic pathogens, such as *Clostridium difficile* infections that often follow antibiotic therapy (Kelly and LaMont, 2008). Fortunately, natural HDPs are rarely found at concentrations high enough to sterilize the environment in their immediate vicinity (Hancock et al., 2016), but this raises the question as to the functions of HDPs within the body. Several pieces of evidence have emerged that natural HDPs may in fact help to shape microbial communities within the host to promote a healthy microbiota, rather than specifically removing pathogenic bacterial species.

Compelling evidence for this idea came from analysis of the microbial communities of cnidarians. Using seven different species of *Hydra* that had been cultivated in the lab under identical conditions for more than three decades, sequencing of the associated microbial community revealed that each species had a distinct microbial community associated with them (Franzenburg et al., 2013). Furthermore, even when co-cultured with another *Hydra* species, the microbial community differences between *Hydra* species did not change, demonstrating that some host-derived factor was dictating the associated bacterial community composition. Arminins are the most highly expressed HDPs in *Hydra* (Augustin et al., 2009) and are only found within the *Hydra* genus. Intriguingly, several orthologs of arminin peptides were found amongst the various *Hydra* species studied and their expression patterns varied depending on the species being evaluated (Franzenburg et al., 2013). Recolonization of germ-free arminin-deficient *Hydra* (with ~50% reduced arminin levels) by donor polyps from other *Hydra* species, revealed that the arminin-deficient *Hydra* were unable to reshape their microbial community into one that resembled their native microbiota, resulting in a distinct microbial community composition. Indeed, the diversity of natural HDP sequences and structures observed for most animals, and even in different body compartments (Nguyen et al., 2011; Mylonakis et al., 2016), might result from species-specific HDPs that have co-evolved to select specific microbial communities beneficial to that specific host, while possibly limiting other species.

The spatial expression pattern of HDPs throughout the body is also known to be tissue and cell type specific and this could well play a role in defining variations in local microbial communities within the same organism. For instance, analysis of HDP expression patterns in the bovine udder revealed differential expression levels of various peptides including the lingual antimicrobial peptide, tracheal antimicrobial peptide, and bovine neutrophil β-defensins 4 and 10 (Tetens et al., 2010). Expression of most of these was confined to the lymph node while *DEFB1* (the β-defensin-1 gene) expression occurred primarily at distal regions of the mammary gland. Furthermore, bovine psoriasin (also known as S100A7) expression was found to be strongly expressed in the streak canal (udder entrance) and the authors suggest that this peptide may prevent the development of coliform mastitis because of its strong antibacterial potency and proximity to the region of the udder that would be exposed to the most pathogens (Tetens et al., 2010). Alternatively, or perhaps in addition, since psoriasin is anti-inflammatory it might serve to prevent inflammation in this environmentally exposed tissue.

In mammals, the role of natural HDPs in maintaining homeostasis within the gut is becoming increasingly appreciated (Bevins and Salzman, 2011; Muniz et al., 2012; Ostaff et al., 2013). For instance, the role of natural HDPs on the host microbiome was evaluated in mice genetically engineered to express human defensin 5 (DEFA5) or lacking the matrix metalloprotease 7 enzyme (MMP7) required to activate the endogenous mouse α-defensins. In both cases, a distinct shift in the composition of the bacterial community was observed, indicating that α-defensins play an important role in shaping the microbiota of the small intestine (Salzman et al., 2010). Specifically, MMP7 deficient mice had low proportions and abundance of *Bacteroides* and mouse intestinal *Bacteroides* (MIB) groups while DEFA5-transgenic mice lacked segmented filamentous bacteria which directly contact the epithelium in small intestines of several animals. In diabetes-prone rats, cathelin-related antimicrobial peptide (CRAMP) expression from β-cells was decreased, suggesting a potential role of this peptide in type 1 diabetes (Pound et al., 2015). At the same time, administration of the human CRAMP homolog, LL-37, to diabetes-prone rats shifted the microbiota toward a composition seen in diabetes-resistant mice (Pound et al., 2015), suggesting that this HDP also plays a role in maintaining gut homeostasis.

As the role of natural HDPs in maintaining homeostasis has become appreciated, their potential to treat microbial dysbiosis has also been considered. HIF-1α is a transcription factor that has been shown to influence the expression of CRAMP in murine myeloid cells (Peyssonnaux et al., 2005). The commensal bacterium *Bacteroides thetaiotamicron* has been shown to activate HIF-1α and promote cathelicidin production in the gut of mice previously exposed to antibiotics which in turn prevented invasive colonization by *Candida albicans* (Fan et al., 2015). This demonstrated that modulation of the mucosal immune effectors might represent a viable therapeutic approach for preventing fungal infections following a course of antibiotic treatment. In this regard, protection against murine *Candida albicans* infections has also been demonstrated using a synthetic immunomodulatory peptide, IDR-1018 (Freitas et al., 2017).

Several HDPs are also produced by epithelial cells within the oral mucosa and the ease of accessibility to this ecological niche within the body has prompted several studies aimed at understanding the relationship of HDPs to oral health. Salivary levels of various HDPs are known to be highly variable within the human population (Tao et al., 2005) and this may be reflective of the diverse oral microbiome composition amongst different individuals (Kilian et al., 2016). Interestingly, low salivary levels of α-defensins (Dale et al., 2006), HNPs 1-3 (Tao et al., 2005), and LL-37 (Davidopoulou et al., 2012) have each been associated with increased levels of caries in children. Patients with morbus Kostmann syndrome, a severe congenital neutropenic disease, also exhibit low LL-37 expression from neutrophils and none of this HDP can be detected in their plasma or saliva (Pütsep et al., 2002). Of note, all patients with morbus Kostmann experience severe periodontal disease, which is consistent with the suggestion that low LL-37 levels in the saliva could contribute to this disease phenotype (Pütsep et al., 2002).

A synthetic peptide C16G2 was developed that was able to specifically target and kill a cariogenic pathogen, *Steptococcus mutans*, within an oral microbial community (Guo et al., 2015). Not surprisingly, when treated with C16G2, the overall microbial community exhibited a dramatic shift possibly due to killing of certain microflora organisms by the peptide or to a reduction in bacterial species that were dependent on *S. mutans* for their maintenance. Importantly, this study demonstrates the possibility of using synthetic peptides to shape and modulate natural microbial communities. Alternatively, synthetic HDPs may prove useful for preventing infections associated with microbes present in complex dental plaque biofilms, such as peptide 1018 that significantly inhibited mixed biofilms formed by natural salivary microflora (Wang et al., 2015b).

The mechanisms by which HDPs maintain this microbial balance within the host are not completely understood, although it is likely that these mechanisms will be dependent on the specific HDP that is expressed at (or delivered to) a given epithelial surface, as well as the type of effector cells in the immediate vicinity that can be influenced by the pleiotropic effects of these molecules. Indeed, it is tempting to speculate that an activity landscape defining the homeostatic activity of HDPs could be exploited to develop prophylactic options to maintain a healthy microbial balance. As our understanding of these processes improves, opportunities to use HDPs as promoters of healthy microflora will surely emerge.

### Diseases Associated With Altered HDP Expression or Activity

Due to their significant role in innate immunity and various inflammatory processes, it is perhaps unsurprising that many diseases and chronic inflammatory conditions have been associated with a dysregulation of the natural HDP response, particularly at epithelial surfaces where natural peptides are present in high abundance or can be induced in response to various environmental stimuli. These include conditions associated with the skin, gut, lungs and several autoimmune disorders (Hancock et al., 2016). Furthermore, there is increasing evidence that natural HDPs can influence tumorigenesis, either positively or negatively depending on the peptide in question and the tissue affected. Many of these topics have been reviewed in detail by others and we will only briefly highlight some of the work that has been described pertaining to several of these conditions.

#### Skin Disorders

The skin is the largest organ in the human body. It is comprised of several different cell types that are organized into a complex architecture that allows skin to perform a wide range of biological functions. Since skin is constantly exposed to bacteria within the environment, one of the main functions of skin is to protect against invading pathogens while maintaining a healthy skin-associated microbiota. Several HDPs have been implicated in skin health and it is not surprising that a dysregulation in these HDP levels can contribute to a variety of skin disorders (Schauber and Gallo, 2008; Marcinkiewicz and Majewski, 2016).

Psoriasis is a relatively common autoimmune disorder characterized by inflamed skin resulting in abnormal skin patches that are itchy, scaly and inflamed. Psoriatic skin is characterized by overexpression of several HDPs and antimicrobial proteins and it is generally thought that the presence of HDPs exacerbates psoriatic lesions (Morizane and Gallo, 2012). Indeed, high β-defensin gene copy number has been associated with increased risk for psoriasis (Hollox et al., 2008) and the human cathelicidin LL-37 is also overexpressed in psoriatic skin (Lande et al., 2007, 2014). Intriguingly, activation of both the innate and adaptive immune response has been implicated in the pathogenesis of psoriasis. For instance, LL-37 has been shown to activate plasmacytoid dendritic cells by breaking tolerance to self-DNA (Lande et al., 2007), while it was demonstrated that LL-37 could also serve as an autoantigen for T-cells (Lande et al., 2014). However, offsetting this is the potent anti-inflammatory activity of LL-37 (Bowdish et al., 2005) which has been clinically tested as a method for counteracting ulcerative lesions (Grönberg et al., 2014). Patients with cutaneous lupus erythematosus also have increased expression of several HDPs which has been proposed to explain why they seldom develop skin infections (Kreuter et al., 2011), although it is unclear how HDP expression contributes to the pathogenesis of this disease in general.

Atopic dermatitis (AD, known colloquially as eczema) is another common inflammatory condition characterized by dry, red and itchy skin. In contrast to psoriasis, however, AD is associated with reduced HDP levels and it has been suggested that impairment of HDP production in AD skin contributes to higher incidence of skin infections, particularly *S. aureus* infections (Marcinkiewicz and Majewski, 2016).

In addition to these inflammatory disorders, a dysregulation of HDP production in chronic wounds has been implicated in a failure of these lesions to heal properly (Haney et al., 2018b). Compounding this issue is that chronic wounds are often colonized by bacteria growing in biofilms (James et al., 2008) that are intrinsically resistant to conventional antibiotics (Lopez et al., 2010) and whose presence may exacerbate inflammation in the wound bed (Zhao et al., 2013). The therapeutic use of natural and synthetic HDPs to promote wound closure while also targeting the bacteria in the biofilm growth state may therefore represent an underexplored strategy to treat chronic wounds (Haney et al., 2018b), and proof of principle has indeed been achieved for venous leg ulcers (Grönberg et al., 2014). Similarly, synthetic peptides have shown efficacy in murine cutaneous abscess infections (Mansour et al., 2016; Pletzer et al., 2017) and sterile skin inflammation mouse models (Wu et al., 2017a).

#### Inflammatory Bowel Diseases

The gastrointestinal tract in humans is home to a large and diverse community of bacteria and other microbes. The ability of the epithelial cells lining the intestinal tract to contain these bacteria is due to the presence of a complex layer of mucus and proteins, including a wide assortment of HDPs (Wehkamp et al., 2007). The term inflammatory bowel disease (IBD) encompasses a range of inflammatory conditions of the intestinal tract. The two most common IBDs are ulcerative colitis, which largely affects the colon, and Crohn's disease, which can affect the entire gastrointestinal tract (Geboes et al., 2018). The exact causes of these conditions are currently unknown but they are likely to involve a combination of genetic, immune and environmental factors. Various HDPs have been implicated in these diseases (Holani et al., 2018), consistent with the potentially important role that these molecules play in regulating overall gut health. For instance, expression of many HDPs is high during colitis, particularly HBD2 and HBD3, and some of these may serve as biomarkers of disease (Wehkamp et al., 2007; Ho et al., 2013). Interestingly, the opposite phenomenon occurs in Crohn's disease, as the levels of several HDPs, including β-defensins and LL-37, are substantially diminished (Wehkamp et al., 2007), which may lead to disruption of the barrier function of the gut and allow bacteria to reach the epithelial cell surface where they elicit an inflammatory response.

#### Lung Disorders

As with other airway epithelial surfaces, the lungs are constantly exposed to bacteria and other molecules that are carried into the lungs with each inhaled breath. Fortunately, our lungs are well-adapted to withstand this constant exposure to potential pathogens and respiratory infections are kept at bay through phagocytes and the mucociliary response. Unfortunately, respiratory infections are relatively common (Vos et al., 2015) and there is evidence that dysregulation of natural HDPs within the lungs can contribute to an increased susceptibility to respiratory infections (Hiemstra et al., 2016). Lung tissue is known to express several HDPs including α-defensins, β-defensins, and LL-37 (Hiemstra et al., 2016), and their expression is often upregulated in response to pathogen exposure, e.g., β-defensin 2 induction in lung cells exposed to *P. aeruginosa* (Harder et al., 2000). Furthermore, CRAMP-deficient mice have been shown to have increased susceptibility to lung infections caused by *Klebsiella pneumonia* (Kovach et al., 2012) implicating a key role for natural HDPs in maintaining healthy lung function.

In addition to preventing respiratory infections, several other inflammatory conditions of the lungs have been associated with a dysregulation of HDP function including: cystic fibrosis (CF), chronic obstructive pulmonary disease (COPD), and asthma. As with the other inflammatory conditions described above, both high and low expression of natural HDPs within the lungs can contribute to various disease pathologies.

CF is an autosomal recessive genetic disorder caused by mutations in the CF transmembrane conductance regulator protein (*CFTR*) gene which regulates anion transport in the airway and other epithelial surfaces (Elborn, 2016). Patients with this disease get mucus buildup within the lungs and have difficulty clearing bacteria which contributes to persistent respiratory infections and chronic inflammation (Elborn, 2016). A direct consequence of this CFTR defect is that the salt concentration within the lungs of CF patients is higher than in healthy individuals (Smith et al., 1996) and this high salt concentration has been shown to inhibit the antibacterial activity of natural HDPs like HNP1 (Turner et al., 1998) possibly contributing to increased susceptibility to bacterial infections. Interestingly, in *in vitro* models, IDR-1018 was able to reduce the exaggerated inflammatory response of CFTR-mutated human airway epithelial cells to bacterial inflammatory agonists, largely by correcting defective autophagosomal clearance (Mayer et al., 2013).

COPD is a progressive lung disease that affects nearly 10% of the population and is particularly prevalent in smokers (Cosio et al., 2009). Patients with COPD have limited and progressively deteriorating lung function and exhibit abnormal inflammatory responses within the small airways and alveoli in their lungs (Cosio et al., 2009). The role of HDPs in COPD has been recognized for some time and the expression of many natural HDPs is often dysregulated in patients afflicted with this condition, which causes patients to have increased lung inflammation and leaves them prone to bacterial infections (Hiemstra et al., 2016). In addition to altered HDP expression, the enzymatic activity of peptidylarginine deiminases (PADIs) has recently been shown to influence the function of natural HDPs in the lungs of smokers with COPD. PADIs are enzymes that postranslationally modify cationic peptidylarginine residues to peptidylcitrulline which blocks their associated cationic charge (Wang and Wang, 2013). Interestingly, the levels of PADI2 are elevated in the lungs of smokers (Makrygiannakis et al., 2008) and recombinant human PADI2 has been shown to citrullinate the Arg residues in LL-37 *in vitro* (Kilsgård et al., 2012). Citrullinated LL-37 exhibits reduced antibacterial activity *in vitro* compared to LL-37 and is more susceptible to protease degradation (Kilsgård et al., 2012), suggesting that this form of the peptide would be less effective and more rapidly cleared from the lungs of COPD patients. More recent work has demonstrated that citrullination of LL-37 also suppressed the immunomodulatory function of this peptide by reducing its anti-inflammatory ability to neutralize the pro-inflammatory activity of bacterial LPS (Koziel et al., 2014), further implicating this process as a contributing factor to COPD progression.

Asthma is the most common inflammatory condition of the lung and, when triggered by dust or allergens, leads to airway inflammation and airflow obstruction (Holgate et al., 2015). The exact cause of asthma is thought to involve a range of environmental and genetic factors; however, one of the features of this diseases is an altered innate immune response (Holgate et al., 2015). Allergic airway inflammation has been shown to suppress innate host defenses in mouse models of asthma, including reducing levels of the mouse cathelicidin CRAMP (Beisswenger et al., 2006). Steroid treatment by glucocorticoids is a common treatment for asthma. However, steroid treatment in a murine model of asthma reduced the levels of pulmonary HDPs and led to increased susceptibility to infections by *P. aeruginosa* (Wang et al., 2013).

Recently, the use of synthetic HDPs was explored as a potential treatment option to overcome the reduced levels of natural HDPs seen in asthmatic lungs. Impressively, subcutaneous administration of IDR-1002 reduced airway hyper-responsiveness in a murine model of house dust mite (HDM) induced allergic asthma (Piyadasa et al., 2018). Mechanistic studies revealed that the peptide suppressed the production of interleukin (IL)-33 in murine lungs and human primary bronchial epithelial cells. Since the levels of IL-33 are often elevated in patients with asthma and disease severity is linked with the levels of this chemokine (Préfontaine et al., 2009), the use of peptide based therapeutics to suppress this key effector molecule represents a potentially unexplored treatment option for asthma. Notably, IDR and HDP peptides have demonstrated activity in a variety of lung infection models including *M. tuberculosis* (Rivas-Santiago et al., 2013a,b) as well as acute and chronic *P. aeruginosa* infection models (Wuerth et al., 2017, 2018), demonstrating anti-inflammatory and/or anti-infective activity.

#### Cancer

The role of natural HDPs in tumorigenesis is complex and not fully understood (for recent reviews, see Droin et al., 2009; Wu et al., 2010; Jin and Weinberg, 2018). Nevertheless, several studies have shown that natural HDPs are dysregulated in various cancers and whether they are purported to promote or prevent cancer progression appears to depend on the type of cancer and which peptide is being considered. For instance, the human cathelicidin LL-37 is expressed in lung (von Haussen et al., 2008), breast (Heilborn et al., 2005), and ovarian (Coffelt et al., 2008) cancers. It has been shown to have angiogenic properties (Salvado et al., 2013) and can serve as a growth factor (Heilborn et al., 2005; von Haussen et al., 2008), two functions which could promote tumor growth *in vivo*. On the other hand, LL-37 has also been shown to kill Jurkat T leukemia cells by inducing apoptosis (Mader et al., 2009) and peptide fragments derived from LL-37 have been identified with direct anticancer activity against several cancer cell lines (Kuroda et al., 2015).

HBD-1 appears to have largely antitumor effects since this peptide is toxic toward late stage prostate cancer cell lines (Bullard et al., 2008), while hBD-1 expression is suppressed in malignant prostate tissue (Donald et al., 2003). Furthermore, four common defensin haplotypes are associated with the increased risk of prostate cancer and high copy numbers of the defensin gene cluster are less observed in prostate cancer patient samples (Huse et al., 2008). Conversely, hBD-3 appears to be carcinogenic as it is highly expressed in cervical cancer (Xu et al., 2016) and carcinomas of the head and neck (Mburu et al., 2011) and has been shown to promote cervical cancer growth in mouse models (Xu et al., 2016).

Based on these examples, it is attractive to speculate that the peptide activity landscape that promotes tumorigenesis is independent of peptides with anticancer properties and it therefore may be possible to specifically enhance the anticancer properties of a peptide as novel chemotherapeutics. In fact, this anticancer activity of HDPs has spurred significant interest into this class of molecules (Gaspar et al., 2013; Felício et al., 2017) as researchers seek to identify and optimize peptides for their direct anticancer effects (Hilchie et al., 2016; Arias et al., 2017).

#### Biofilm-Associated Infections

While this category is not *per se* a specific disease type, biofilms form locally and can be associated with a variety of pathological circumstances (including some of those described above). The seminal observation that the human cathelicidin LL-37 inhibited biofilm growth at sub-inhibitory concentrations (Overhage et al., 2008) revealed that HDPs could potentially be exploited as novel antibiofilm agents. Many more antibiofilm peptides have since been identified from screening available synthetic peptide libraries for biofilm specific activity (de la Fuente-Núñez et al., 2014a,b; Reffuveille et al., 2014) or using activity-guided design strategies to optimize known antibiofilm peptide sequences (Haney et al., 2015). Indeed, the antibiofilm activity of newly described HDP sequences is now often reported in addition to the standard MIC values.

The reason for the preferential activity of some peptides against biofilms (Overhage et al., 2008; de la Fuente-Núñez et al., 2014b, 2015) is likely related to differing abilities to target the physiological underpinnings of biofilms as a stress-coping state, such as attacking the (p)ppGpp nucleotide signals that mediate the stringent stress response (Potrykus and Cashel, 2008). This suggests that the mechanism of action (and by extension, the activity landscape) of HDPs with antibiofilm activity is independent from the cellular functions that target planktonic bacterial cells. Given that overall HDPs are seen as “dirty drugs” (Hancock and Sahl, 2006), it is probable that multiple mechanisms of action occur downstream of the stress response, likely dependent on the environment of the HDP and the composition of the biofilm itself. Whether or not these mechanisms are truly independent or interdependent remains to be determined.

In an effort to begin to appreciate the range of HDP sequences with antibiofilm activity, an online database has been established to collect information on peptide sequences with documented activity specifically directed toward bacterial biofilms (Luca et al., 2015). In addition, a computational approach using QSAR modeling was recently used to identify novel antibiofilm specific peptides with therapeutic potential (Haney et al., 2018a). Using antibiofilm activity data derived from a SPOT-synthesized peptide arrays consisting of singly-substituted variants of 1018, a QSAR model was generated to describe this antibiofilm activity. The resulting model, which identified the seven most important molecular descriptors from a starting list of ~2,500 descriptors, was able to accurately predict 85% of the antibiofilm peptides within the training set. This QSAR model was subsequently used to predict potential antibiofilm peptides *in silico* from a virtual library consisting of 100,000 peptides and a subset were synthesized to evaluate and confirm their antibiofilm activity *in vitro* and *in vivo* (Haney et al., 2018a).

As more diverse antibiofilm peptide sequences are reported with greater potency, the activity landscape of antibiofilm specific peptides will begin to materialize. As details regarding their mechanism of action and overlapping activity landscapes with other biological functions are appreciated, multifunctional peptides capable of exerting an array of biological effects are sure to emerge as promising drug candidates to treat biofilm-associated infections.

## Emerging Concepts in HDP Research

**Figure 2:**
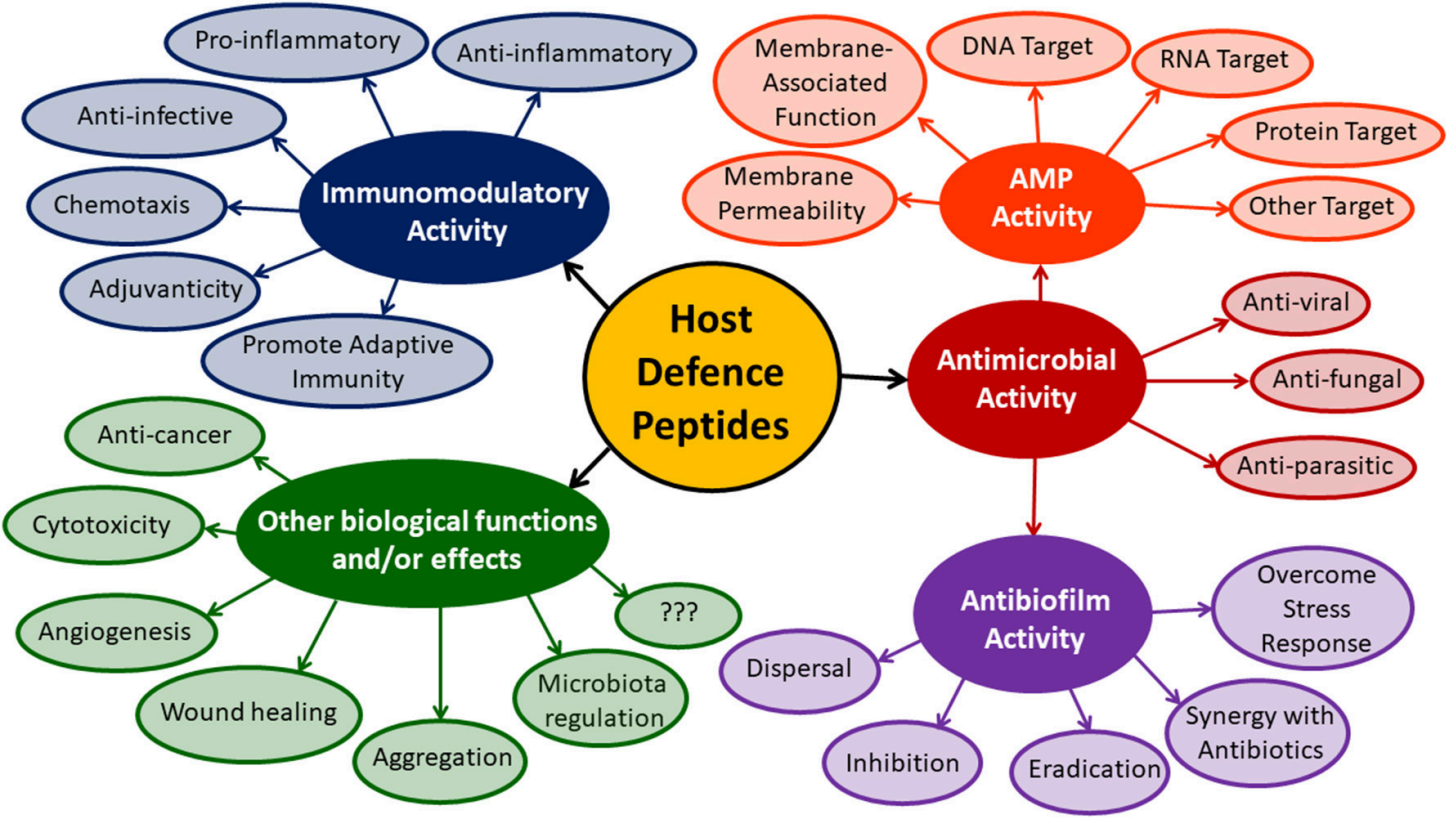
Diversity of biological functions described for HDPs.

In this review, we have highlighted several biological functions that have been reported for natural and synthetic HDPs. The breadth and diversity of these activities is vast (Figure 2) and new peptide sequences and biological functions are continuously being reported in the literature. Indeed, the majority of new studies continue to focus on the antibacterial effects of HDPs with an emphasis on membrane-active peptide sequences. However, the plethora of other activities described for HDPs deserves increased appreciation and detailed mechanistic studies that push beyond the idea of “membrane busters” will be necessary to finally unlock the therapeutic potential of these biomolecules. In an ideal world, synthetic HDPs could be designed to maximize a desired biological function provided sufficient data existed to accurately define the activity landscape of all possible activities of interest. At the moment, defining these activity landscapes and accurately mapping their chemical space is a daunting challenge, but with new sophisticated screening and modeling techniques, it is something that can likely be overcome.

An important consideration that is often overlooked in many optimization studies is whether the assay conditions used to measure a biological property of an HDP accurately capture the behavior of the peptide that would occur *in vivo*. For instance, measuring the MIC of a peptide in phosphate buffer or nutrient limiting conditions will often yield values that appear more potent than MICs recorded in rich media or in the presence of high salt (Mahlapuu et al., 2016). Furthermore, the presence of host cells can also interfere with the observed potency of AMPs. For instance, preincubation of several AMPs with red blood cells (RBCs) drastically reduced their antibacterial efficacy against *E. coli* and *S. aureus in vitro* (Starr et al., 2016). However, when added to a cell suspension containing both RBCs and bacteria, the inhibition of antimicrobial activity was not observed (Savini et al., 2017), highlighting the influence of experimental setup on the observed biological activities. In practice, the optimization of an HDP actually enhances peptide sequences for a very specific functional assay. The more faithfully that an experimental setup represents conditions that would be encountered *in vivo*, the greater the likelihood that the optimized synthetic HDPs would retain their biological functions *in vivo*.

It is also presently unclear whether a peptide should or could be simultaneously optimized for multiple biological functions or if a specific biological activity should be the driving force that guides the optimization strategy. For instance, the antibacterial and antibiofilm properties of HDPs appear to be independent of one another (Overhage et al., 2008; de la Fuente-Núñez et al., 2014b; Haney et al., 2018a) and they are likely defined by distinct activity landscapes. This is also probably the case for AMPs and immunomodulatory functions (Scott et al., 2007) as well as antibiofilm, chemokine induction and anti-inflammatory activities (Haney et al., 2015). Perhaps ultimately the best candidate peptide will be one that has the best compromise of a mixture of activities. Furthermore, HDPs like LL-37 exert their pleiotropic effects on the host through binding to various receptors or intracellular targets, as well as interacting with the cell membrane (Verjans et al., 2016). Presumably, each of these functions occurs because of a specific interaction between LL-37 and a particular biomolecule but whether the enhancement of a unique HDP interaction could be teased apart to target a specific immune cell or signaling pathway of interest remains to be elucidated.

A long recognized (Scott et al., 1999b) but increasingly appreciated (Lewies et al., 2018) ability of HDPs to synergize with conventional antibiotics holds promise as a means to overcome specific bacterial resistance mechanisms or restore the antibacterial potency of previously effective antibiotics. Several AMPs have been shown to synergize with conventional antibiotics *in vitro* (Choi and Lee, 2012; Mataraci and Dosler, 2012; Hwang et al., 2013; Gier et al., 2016; Wu et al., 2017b) and these protective effects have been demonstrated in *in vivo* infection models (Otvos et al., 2018) providing an exciting path forward for the development of AMPs as adjunctive therapies for conventional antibiotics. This combination approach can be applied to bacteria growing within biofilms as antibiofilm peptides have been shown to synergize with conventional antibiotics to prevent and eradicate biofilms *in vitro* (Dosler and Karaaslan, 2014; Reffuveille et al., 2014; de la Fuente-Núñez et al., 2015). Recent studies *in vivo* revealed that this synergy can be extended to hard-to-treat cutaneous abscesses in mice and that peptides could be used in combination with an array of antibiotics to effectively reduce the size of abscesses caused by all of the ESKAPE pathogens (Pletzer et al., 2018). The mechanism of antimicrobial synergy was proposed to involve promoting antibiotic penetration and disruption of the stringent response. Thus, future design studies could be aimed at promoting specific aspects of this synergistic relationship to further enhance the effectiveness of drug combinations.

Enhancing endogenous expression of natural HDPs as a therapeutic approach has also been a topic of considerable interest in recent years. Much of this work has stemmed from the observation that transcription of the *CAMP* gene is regulated by the vitamin D receptor (Gombart et al., 2005; Liu et al., 2006) and it has been shown that vitamin D levels directly correlate with LL-37 levels in healthy individuals (Bhan et al., 2011; Dixon et al., 2012). Clinical trials have examined the effects of supplementing patients suffering from a variety of inflammatory disorders with vitamin D in an effort to enhance LL-37 levels and promote innate immune functions associated with this peptide. The benefits of such a therapeutic approach have been seen in several diseases including: CF (Grossmann et al., 2012), atopic dermatitis (Hata et al., 2008), cirrhosis (Zhang et al., 2016), tuberculosis (Coussens et al., 2012), and Crohn's disease (Raftery et al., 2015). The success of vitamin D as an inducer of a natural HDP (although it should be mentioned that this vitamin has many other immunomodulatory properties), has spurred research looking to identify other compounds capable of the same effect. For instance, butyrate and other short chain fatty acids are well-known inducers of LL-37 expression (Schauber et al., 2003; Jiang et al., 2013). Proteins and biomolecules produced by commensal bacteria have also been shown to modulate expression of HDP levels in the host. For example, a bacterial lipoprotein from the commensal bacterium *Fusobacterium nucleatum* called FAD-I (Fusobacterium Associated Defensin Inducer) was recently shown to activate hBD-2 expression in oral epithelial cells (Ghosh et al., 2018). Since *F. nucleatum* is resistant to direct killing by hBD-2, it was speculated that this may represent a co-evolution of a commensal organism with the human host to outcompete bacteria that would be susceptible to this HDP.

Finally, there are several perceived limitations to the development of HDPs as viable therapeutics that warrant some discussion. The most often cited issues associated with peptide drugs include high production costs, low stability and bioavailability *in vivo* as well as the potential to induce an immunogenic response (Marr et al., 2006; Vlieghe et al., 2010). Issues associated with production costs are likely unfounded as these can be addressed by optimizing large scale synthesis procedures (Bray, 2003). With regards to bioavailability, peptides appear to have unusual pharmacokinetics when delivered systemically, with a rapid initial distribution blood followed by moderate stable levels appearing in various tissues for up to 4 (Bolouri et al., 2014) or 6 h (Brunetti et al., 2016). These concentrations may be too low to achieve direct antibacterial activity (Roversi et al., 2014) but they could prove useful in situations where these levels are sufficient (e.g., as immune modulators), or certain peptides may delivered locally to achieve high concentrations in the affected tissue (e.g., for skin or lung infections). In general, small therapeutic peptides are considered to be non-immunogenic (McGregor, 2008); however, detailed studies on the ability of synthetic HDPs to elicit an immunogenic response are largely lacking. In our experience, generating antibodies against synthetic HDPs is difficult, suggesting that HDPs may occupy an immunological “blindspot” (perhaps mediated by clonal T-cell deletion during development) with regards to adaptive immunity. Synthetic HDPs also have potential issues associated with toxicity mediated in part by a tendency to aggregate in the presence of specific anions and body fluids (Haney et al., 2017) or through non-specific interactions with host cells that cause cell lysis. Formulating peptides with various delivery vectors such as liposomes (Yang et al., 2011; Allen and Cullis, 2013), nanoparticles (d'Angelo et al., 2015; Nordström and Malmsten, 2017; Wadhwani et al., 2017) or covalently attaching them to bio-compatible polymers (Sahariah et al., 2015; Pranantyo et al., 2016; Kumar et al., 2017) can potentially mitigate some of these effects while retaining the desired biological functions, but further studies will be required to define how a given peptide-drug formulation combination impacts the pharmacokinetics, pharmacodynamics, and activity profile of a given therapeutic peptide.

## Concluding Remarks

Faced with the prospect of a world without effective antibiotics, it is imperative that we continue the search for new anti-infective strategies and especially alternatives to conventional antibiotics. AMPs and HDPs have been championed as candidate drugs that could fill the void created by the rise of antibiotic resistance, largely by considering them as a new wave of antibiotics. Unfortunately, even after nearly 40 years of work since their discovery, we have yet to see this promise fulfilled. It could be argued that the writing is on the wall for AMPs and that we will never make these compounds into viable drugs. However, we maintain that HDPs overall still have tremendous potential as therapeutic options for bacterial infections but we have been focusing our efforts on mapping the wrong activity landscape related to anti-infective activity. The multi-faceted nature of HDPs and their ability to influence a wide range of biological processes opens the door to expanding our understanding of other activity landscapes within the chemical space of HDPs. As our understanding of these other activity types improves, and the mechanistic details underpinning these other processes are laid bare, this will undoubtedly lead to the development of HDP based drugs that are effective against infectious diseases as well as inflammatory conditions. Indeed, the antibacterial mountain of HDPs has probably been conquered, but the exploration of the peaks and valleys that make up the entire chemical landscape of HDPs has only just begun.

## Author Contributions

EH and SS wrote the first draft of the manuscript. All authors contributed to manuscript revision, approved the final version and contributed to the conception of this work.

### Conflict of Interest Statement

HDPs, including some of those described here but published as open source articles, have been filed for patent protection by RH and EH and assigned to their employer the University of British Columbia, and licensed to ABT Innovations Inc., Victoria, Canada, in which the University of British Columbia, EH and RH own shares. The remaining author declares that the research was conducted in the absence of any commercial or financial relationships that could be construed as a potential conflict of interest.
